# Surgical Margins After Computer-Assisted Mandibular Reconstruction: A Retrospective Study

**DOI:** 10.3389/froh.2021.806477

**Published:** 2022-01-13

**Authors:** Erika Crosetti, Giovanni Succo, Bruno Battiston, Federica D'Addabbo, Martina Tascone, Elena Maldi, Ilaria Bertotto, Mattia Berrone

**Affiliations:** ^1^Head Neck Oncology Unit, Candiolo Cancer Institute, Fondazione del Piemonte per - l'Oncologia-Istituto di Ricovero e Cura a Carattere Scientifico (FPO-IRCCS), Candiolo, Italy; ^2^Department of Oncology, University of Turin, Orbassano, Italy; ^3^Department of Orthopedics and Traumatology, Hand and Microsurgery Unit, Orthopedic and Trauma Centre, Azienda Ospedaliero Universitaria (AOU) Città Della Salute e Della Scienza, Turin, Italy; ^4^Pathology Unit, Candiolo Cancer Institute, Fondazione del Piemonte per - l'Oncologia-Istituto di Ricovero e Cura a Carattere Scientifico (FPO-IRCCS), Candiolo, Italy; ^5^Radiology Unit, Candiolo Cancer Institute, Fondazione del Piemonte per - l'Oncologia-Istituto di Ricovero e Cura a Carattere Scientifico (FPO-IRCCS), Candiolo, Italy

**Keywords:** fibular free flap, mandibular reconstruction, virtual surgical planning, surgical margins, oral cancer

## Abstract

**Purpose:** The use of virtual surgical planning in head and neck surgery is growing strongly. In the literature, its validity, accuracy and clinical utility for mandibular reconstruction are widely documented. Virtual planning of surgical bone resection and reconstruction takes place several days before surgery and its very sensitive nature can negatively affect an intervention aimed at maximum precision in term of oncological safety.

**Methods:** The study focuses on a retrospective evaluation of the surgical margins in 26 consecutive cases with oral cavity malignancy and who underwent computer-assisted mandibular resection/reconstruction guided by the different types of bone, periosteal and peri-mandibular tissue involvement. The goal was to analyze the strategic and technical aspects useful to minimize the risk of positive or close margins and to vary the reconstructive strategy in the case of intraoperative findings of a non-radical planned resection.

**Results:** No intraoperative or perioperative complications occurred. In 20 patients, virtual surgical planning permitted mandibular reconstruction to be performed using composite fibular free flaps, characterized by high accuracy and negative bone margins. In the remaining 6 patients, also virtually planned but otherwise reconstructed due to poor general condition (advanced age, severe comorbidity), negative bone margins were obtained. Intraoperative enlargement of the resection was carried out in one case and positive soft tissue margins were observed in another case.

**Conclusion:** The results were satisfactory in terms of oncological radicality and precision. The functional benefits and reduction in operating times, previously demonstrated in other articles also by the authors, seem to justify the side effects related to the risk of modifying the planned surgery. During virtual planning, the surgeons must bear in mind that an unexpected progression of the tumor or a limited planned resection will entail modifying the extent of the resection intraoperatively and nullifying the virtual planning on which the reconstruction was based. Further investigations are necessary to clarify all aspects of virtual surgical planning in this setting.

## Introduction

Surgery represents the first-choice therapy for oral cavity squamous cell carcinoma (OCSCC). Surgical approaches and techniques are influenced by the three-dimensional nature of the anatomic site and the tissues present as well as the progression pathways of the disease. To be effective, surgery must be achieved with free margins of at least 1 cm in all directions if possible. Nevertheless, the percentage of positive/close margins is quite high, ranging from 30 to 65% in different series [1–3].

In oral oncology, the clinical significance of surgical margins on both bone and oral mucosa has always aroused great scientific interest [4] and are a consistent prognostic factor [5]; adequate resection margins in OCSCC lead to a higher rate of survival and an important reduction in local recurrence [1], while inadequate resection results in the need for adjuvant therapy. OCSCC involving the mandible, directly or contiguously, determines the need for a marginal or segmental resection of the jaw. If the patient is not affected by important comorbidities, primary reconstruction using free flaps is considered the gold standard worldwide; among these solutions, the most practiced is reconstruction using a fibular free flap (FFF) [6, 7]. A primary reconstruction generates significant benefits for a patient's residual quality of life, avoiding major surgical procedures for secondary reconstruction and often allowing a fixed dental prosthesis to be used [8]. Virtual resection/reconstruction planning (computer-assisted mandibular reconstruction—CAMR) [9, 10] has established itself as an effective technique to reduce operating times and achieve millimeter precision in modeling the revascularized bone replacing the mandible. The design process takes place several days before surgery. This is a sensitive procedure that can negatively affect an intervention aiming for maximum precision, made more difficult due to the added risk of planning an incomplete resection, resulting in positive or close margins. Therefore, additional more in-depth knowledge is required regarding the safety of the procedure in terms of surgical margins, especially at the bone level.

The primary aim of this study was to retrospectively evaluate the oncological safety and the accuracy of virtual surgical planning in patients affected by oral malignancies, with direct/suspected mandibular involvement and reconstructed with free flap. The secondary end-point was to discuss any technical solutions useful to minimize the risk of insufficient resection and/or to change the strategy during surgery due to an unexpected finding of tumor extension.

## Materials and Methods

### Study Population

A retrospective study was carried out on the use of virtual surgical planning, using the CAMR technique, repositioning technique (REP-TECH) and computer-assisted rim mandibulectomy (CARM) or stereolithographic models in the reconstruction of patients affected by squamous cell carcinoma of the oral cavity who were submitted to segmental or rim-mandibulectomy and primary reconstruction with a free flap (fibular free flap—FFF, radial forearm free flap—RFFF).

The study cohort (Group A) included 26 consecutive patients treated in the period from January 2014 to October 2020 in the Head and Neck Oncology Unit at the FPO IRCCS, Candiolo Cancer Institute, applying the CAMR technique, repositioning technique (REP-TECH) and computer-assisted rim mandibulectomy (CARM).

The control group (Group B) included 21 consecutive patients treated in the period from January 2006 to October 2013 in the ENT Department of Turin, Martini Hospital and Sal Luigi Gonzaga Hospital, operated with the aid of stereolithographic models.

Demographic data for the patients of Group A are summarized in Table 1 and those in the group B in the Table 2. All of the procedures were conventional in terms of technique and indications, in accordance with the current guidelines and therefore also in accordance with the ethics standards of the Institutional and/or National Research Committee and with the 1964 Helsinki Declaration and its later amendments. The advantages and disadvantages of this approach as well as the alternative approaches were clearly and fully explained to the patients when seeking informed consent for the procedures. All patients underwent the same clinical assessment during the 3 weeks before surgery including clinical examination, nutritional status evaluation [body mass index (BMI)], biopsy/pathological examination, maxillofacial and neck MRI/CT scan (thin slice CT scan ≤ 1 mm), and total body PET scan. Three surgeons (G.S., E.C., and B.B.) carried out all of the procedures.

**Table 1:** Demographic data for Group A (26 patients).

**Characteristics**	**No. of patients (%)**
Age, years	
Mean	59
Range	Male 33–78
	Female 43–78
Sex	
Male	14 (53.8)
Female	12 (46.2)
Risk factors	
Non smoker	11 (42.3)
Previous smoker	9 (34.6)
Smoker	6 (23.1)
Pre-treatment	
Yes	10 (38.5)
No	16 (61.5)
Site	
Retromolar trigone	9 (34.6)
Alveolar crest	6 (23.1)
Floor of the mouth	6 (23.1)
Hemimandible	3 (11.5)
Mandibular symphysis	2 (7.7)
Histology	
Squamous cell carcinoma	24 (92.4)
Verrucous carcinoma	1 (3.8)
Bone metastases from breast cancer	1 (3.8)

**Table 2:** Demographic data for Group B (21 patients).

**Characteristics**	**No. of patients (%)**
Age, years	
Mean	56
Range	Male 31–73
	Female 37–75
Sex	
Male	15 (71.4)
Female	6 (28.6)
Risk factors	
Non smoker	1 (4.8)
Previous smoker	4 (19)
Smoker	16 (76.2)
Pre-treatment	
Yes	8 (38)
No	13 (62)
Site	
Alveolar crest	3 (14.3)
Hemimandible	15 (71.4)
Mandibular symphysis	3 (14.3)
Histology	
Squamous cell carcinoma	21 (100)

Inclusion criteria were as follows: patients with oral cavity proven squamous cell carcinoma with or without clinically metastatic lymph nodes cN0/N+, candidates for free flap reconstruction.

Exclusion criteria included: patients without oral malignancies or patients not candidate for flap reconstruction for important comorbidities.

### Surgical Procedure

**Figure 1:**
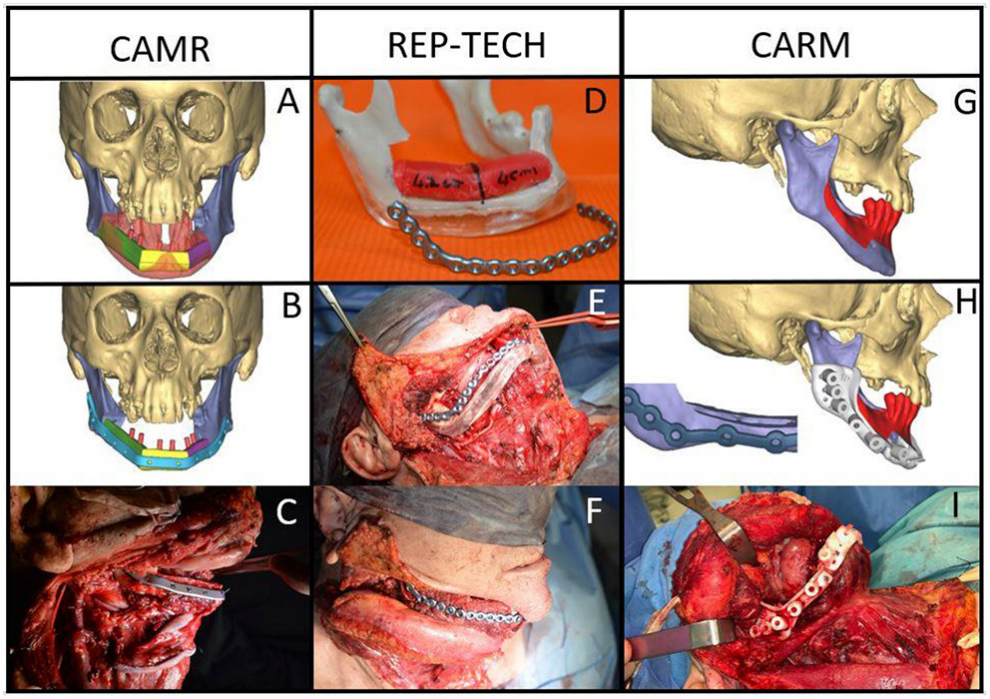
Malignant neoplasms in the mandible with bone involvement: CAMR: **(A,B)** virtual resection and reconstruction program; **(C)** the fibula-plate complex fixed on native mandibular bone; REP-TECH: **(D)** virtual resection and reconstruction program translated onto a stereolithographic model with repositioning template and reconstructive plate; **(E)** repositioning template and reconstructive plate fixed on the native mandible; **(F)** the fibula-plate complex fixed on the native mandibular bone; CARM: Mandible malignant neoplasms with marginal bone involvement: **(G)** virtual resection program (rim-mandibulectomy); **(H)** marginal mandibulectomy cutting guide and 3D printed reinforcement customized titanium plate; **(I)** marginal mandibulectomy cutting guide fixed on the mandible.

All patients of the Group A underwent segmental mandibulectomy or marginal mandibulectomy. The resection and reconstruction were virtually planned, and surgical procedures were performed with the aid of bone cutting guides. The bone resection was planned with a distance of at least 1 cm from the radiologically visible lesion. In most cases, further clinical evaluation was carried out during the virtual planning to avoid as far as possible the risk of underestimating the extent of the tumor in the peri-mandibular tissues. The following three computer-assisted programming techniques were adopted: computer-assisted mandibular reconstruction (CAMR) [9, 10], repositioning technique (REP-TECH) [11] and computer-assisted rim mandibulectomy (CARM) (Figure 1).

All the patients of the Group B underwent segmental mandibulectomy or marginal mandibulectomy and FFF reconstruction planned with the aid of stereolithographic models.

### Pre-operative Planning

CAMR consists in virtual resection resective and reconstructive program using specialized software, that also aids the production of customized cutting guides and plates using CAD-CAM technology.

REP-TECH is a technique for fibular free flap modeling and repositioning, after segmental resection of the mandible. The technique entails the preoperative preparation of a resin repositioning template, surgical cutting guides, and manual pre-shaped reconstructive plate on a stereolithographic model; the measurements of bone resection are carried out virtually and subsequently transferred to the stereolithographic model.

CARM uses the same method as CAMR, allowing the production of cutting guides for precise marginal mandibulectomy and a pre-shaped plate to reinforce the residual mandible.

The stereolithographic models of the mandible provide an accurate three-dimensional replica of the patient's mandible, allowing to bend the titanium plate pre-operatively, resulting in a positive impact on the accurancy of the reconstruction and a reduction in surgical time [12].

The surgical margins were evaluated using pathology reports. The time elapsing from CT scan to the supply of the material and then to surgery, the bone invasion pattern, and distances of bone margins from the radiologically visible lesion were all recorded and analyzed.

## Results

### Patient Demographic Background

#### (Group A)

Twenty-six patients (14 men and 12 women) with an overall average age of 59 years (men: age range, 33–78; women: age range, 43–78) affected by malignant neoplasms with bone and/or peri-mandibular soft tissue and periosteal invasion were surgically treated. Ten patients were pre-treated (seven patients by transoral surgery, three patients by multi-modal therapy (surgery + radiation therapy). The histological examination on biopsy reported 24 OCSCC, one verrucous carcinoma and one mandibular metastasis from breast cancer. Imaging showed a mandibular bone infiltrative pattern in 20 cases (11 men, nine women) and erosive pattern in six cases (three women, three men).

The virtual planning consisted of 15 CAMR (nine men, six women), nine REP-TECH (four men, five women), and two CARM (one man, one woman).

#### (Group B)

Twenty-one patients (15 men and six women) with an overall average age of 56 years (men: age range, 31–73; women: age range, 37–75) affected by malignant neoplasms with bone and/or peri-mandibular soft tissue and periosteal invasion were surgically treated. Eight patients were pre-treated (six patients by transoral surgery, two patients by multi-modal therapy (surgery + radiation therapy). The histological examination on biopsy reported 21 OCSCC. Imaging showed a mandibular bone infiltrative pattern in 15 cases (12 men, three women) and erosive pattern in six cases (three women, three men).

### Reconstructive Procedures

#### (Group A)

The bone resection was virtually planned in all procedures. In 20 patients, mandibular reconstruction was performed by an osteo-fascio-cutaneous FFF (12 CAMR, eight REP-TECH). Due to poor general condition (advanced age, severe comorbidity), patients were reconstructed using a reconstructive plate wrapped with a pectoralis major flap (three CAMR, one REP-TECH). two patients underwent CARM with customized mandibular reinforcement plates and a radial forearm free flap for soft tissue reconstruction (RFFF). The average time elapsed between the CT scan and the delivery of the material necessary for the three different techniques was 19 days (CAMR range 8–31 days, CARM 21–30 days, REP-TECH 8–10 days). The average time from CT scan to surgery was 29.5 days (CAMR range 18–42 days, CARM range 26–38 days, REP-TECH range 8–40 days).

OCSCC was confirmed in 24 patients; in one patient a verrucous carcinoma was diagnosed and in another one, a mandibular metastasis from breast cancer.

Soft tissues negative margins (> 5 mm) were obtained in 17 cases (65%), close (= or <5 mm) in 8 cases (31%) and positive in only one case (4%). Periosteal invasion was histologically confirmed in all 26 patients: the lateral periosteal margins were negative in the whole series. Bone invasion was confirmed in 20 cases (77%). Out of these 20 patients, 17 showed an infiltrative pattern and three an erosive pattern at imaging (Table 3). In six patients (23%), no bone invasion was found by the pathologist, although there were elements of radiological and clinical suspicion. Three patients had severe periodontal disease near the tumor thus simulating bone invasion [13]. In these patients, the bone lesions turned out to be large osteitic areas associated with dental problems, however, periosteal invasion was histologically highlighted. Two patients showed mandibular bone reabsorption: marginal mandibulectomy was necessary due to periosteal invasion and the final pathological report confirmed an OSCC coming close to the mandibular bone and involvement of the periosteum, without microscopic evidence of infiltration but with marked signs of bone remodeling. One patient was treated due to a large metastatic lesion from breast cancer.

**Table 3:** Histological data for the Group A (26 patients).

***N***	**cTNM (TNM VIII Eds)**	**pTNM (TNM VIII Eds)**	**Virtual surgical planning technique**	**Type of reconstruction**	**Resection margins**	**Vascular embolization**	**Perineural invasion**	**Bone invasion**	**Bone margins**	**Periosteal margins**	**Soft tissue margins**	**Radiological pattern**	**Adjuvant treatments**	**F-UP (16–84 months)**
1	rT4aN3b	ypT4aN2b	CAMR	FFF	Close	No	Yes	Yes	Negative	Negative	Close (3 mm)	Infiltrative	CRT	DWD
2	rT4N2b	ypT3N0	CAMR	FFF	Negative	No	No	No	Negative	Negative	Negative	Infiltrative	None	NED
3	cT4N2b	pT4aN2b	CAMR	FFF	Close	Yes	Yes	Yes	Negative	Negative	Close (1 mm)	Erosive	CRT	NED
4	cT3N3b	pT4aN2b	CAMR	FFF	Negative	No	Yes	Yes	Negative	Negative	Negative	Infiltrative	CRT	DWD
5	cT3N2b	pT4aN3b	CAMR	FFF	Close	Yes	Yes	Yes	Negative	Negative	Close (1.5 mm)	Infiltrative	CRT	DOD
6	cT4aN1	pT4aN0	CAMR	CRP+PM	Close	No	Yes	Yes	Negative	Negative	Close (4 mm)	Erosive	CRT	NED
7	cT4aN0	pT4aN0	CAMR	FFF	Negative	No	Yes	Yes	Negative	Negative	Negative	Infiltrative	RT	NED
8	cT2N2a	pT2N0	CARM	CARM + RFFF	Negative	No	No	No	Negative	Negative	Negative	Erosive	None	NED
9	rT4aN0	ypT4aN0	REP-TECH	MRP+PM	Negative	No	Yes	Yes	Negative	Negative	Negative	Infiltrative	RT	NED
10	rM1 (breast cancer)	ypM1	CAMR	FFF	Negative	No	No	Yes	Negative	Negative	Negative	Infiltrative	CT	NED
11	cT4aN2b	pT4aN2c	CAMR	FFF	Negative	Yes	Yes	Yes	Negative	Negative	Negative	Infiltrative	CRT	NED
12	rTxN3	yTx N3b	REP-TECH	FFF	Close	Yes	Yes	Yes	Negative	Negative	Close (1 mm)	Infiltrative	CRT	DWD
13	cT4aN1	pT4aN2a	CAMR	FFF	Negative	Yes	No	Yes	Negative	Negative	Negative	Infiltrative	CRT	DWD
14	cT4a N0	pT4aN1	REP-TECH	FFF	Negative	No	Yes	Yes	Negative	Negative	Negative	Infiltrative	RT	DOD
15	rT4aN0	ypT4aN0	CAMR	FFF	Negative	No	Yes	Yes	Negative	Negative	Negative	Infiltrative	RT	NED
16	cT4aN0	pT4aN0	REP-TECH	FFF	Negative	No	Yes	Yes	Negative	Negative	Negative	Infiltrative	RT	DWD
17	rT3N1	ypT2N1	CAMR	FFF	Negative	Yes	Yes	No	Negative	Negative	Negative	Erosive	CRT	NED
18	cT4aN2	pT4aN2c	REP-TECH	FFF	Close	Yes	Yes	Yes	Negative	Negative	Close (1 mm)	Infiltrative	CRT	DWD
19	rcT4aN1	ypT4aN1	REP-TECH	FFF	Positive	Yes	No	Yes	Negative	Negative	Positive	Infiltrative	CRT	NED
20	cT3N1	pT3N1	REP-TECH	FFF	Negative	No	Yes	No	Negative	Negative	Negative	Infiltrative	CRT	NED
21	rT4aN0	ypT4aN0	CAMR	FFF	Negative	Yes	Yes	Yes	Negative	Negative	Negative	Infiltrative	RT	DOD
22	cT4aN1	pT4aN1	REP-TECH	FFF	Negative	No	No	Yes	Negative	Negative	Negative	Infiltrative	CRT	DWD
23	cT3N0	pT3N0	CARM	CARM + RFFF	Close	No	No	No	Negative	Negative	Close (4 mm)	Erosive	RT	NED
24	rcT4N0	ypT4N0	CAMR	CRP+PL	Close	No	No	Yes	Negative	Negative	Close (4 mm)	Erosive	CRT	NED
25	cT4aN0	pT4aN0	REP-TECH	FFF	Negative	Yes	No	Yes	Negative	Negative	Negative	Infiltrative	RT	DWD
26	cT4aN1	pT4aN3b	CAMR	CRP+PM	Negative	Yes	Yes	Yes	Negative	Negative	Negative	Infiltrative	CRT	NED

*CARM, computer-assisted rim mandibulectomy; CAMR, computer-assisted mandibular reconstruction; REP-TECH, repositioning technique; FFF, free fibula composite flap (bone + soft tissue); CRP + PM, customized reconstructive plate + pectoralis major flap; MRP + PM, manually pre-shaped reconstructive plate on stereolithographic models + pectoralis major flap; CARM + RFFF, computer assisted rim mandibulectomy + radial forearm free flap; CRT, concurrent chemoradiation; RT, radiotherapy; NED, non-evidence disease; DWD, dead for disease; DOD, dead for other disease*.

**Figure 2:**
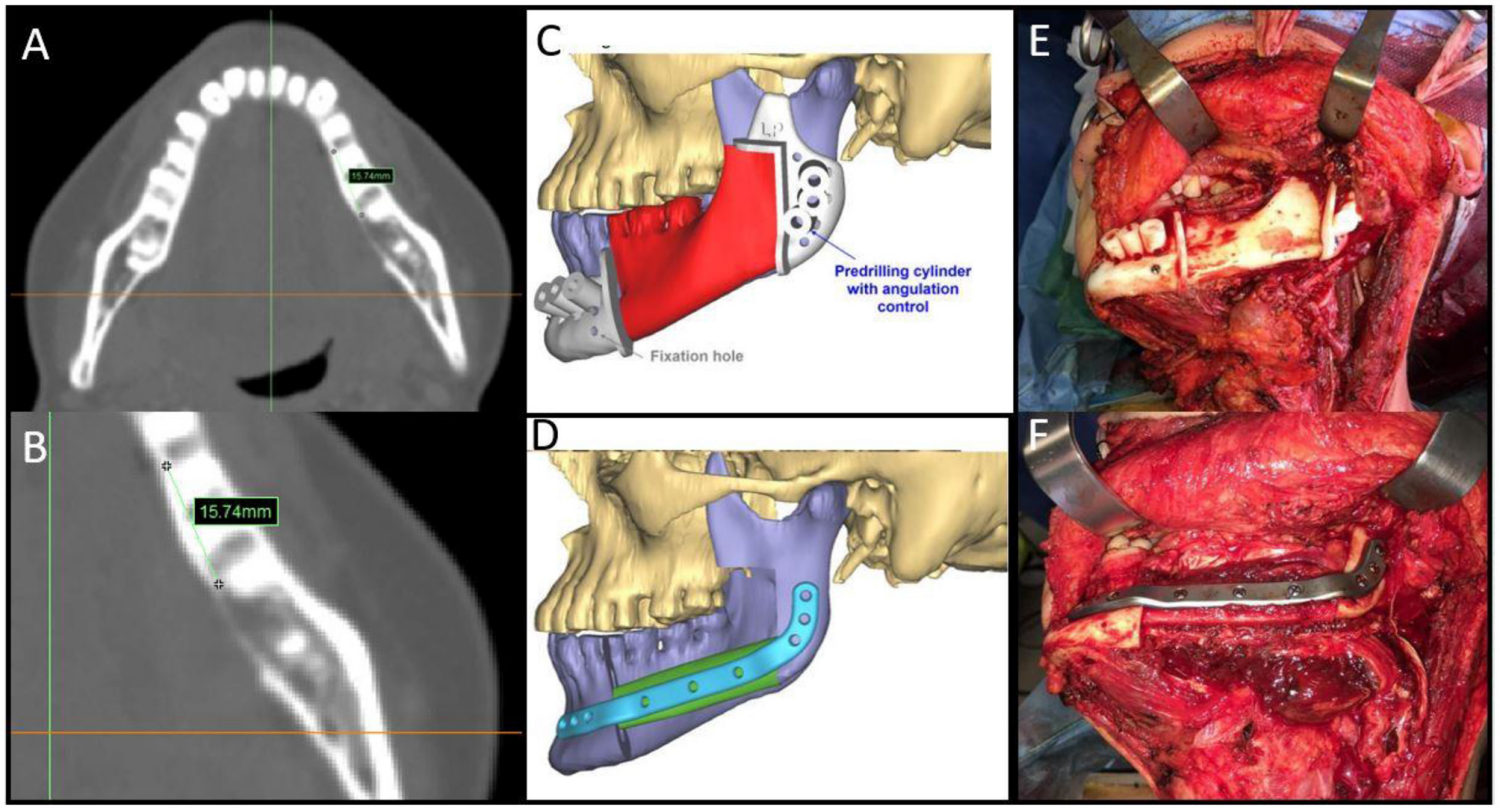
**(A,B)** Malignant neoplasms in the mandible with bone involvement showing measurement details from the radiologically visible bone involvement; **(C,D)** CAMR virtual resection and reconstruction program; **(E)** mandibular cutting guides fixed on the mandible; **(F)** the fibula-plate complex fixed on native mandibular bone.

To prevent positive bone margins, the mean distance from the virtual bone margins to the radiologically visible lesion was 1.3 cm for infiltrative bone lesions (range from 1 to 1.5 cm) and 1 cm for erosive lesions (range from 1 to 1.3 cm). In the latter case, osteotomy was performed to 1 cm from the virtual bone margin (Figure 2). In 25 patients, the virtual planning made it possible to reach free bone margins, clinically evaluated during surgery, without changing the planned resection. In one patient, it was necessary to modify the planned surgery intraoperatively due to periosteal invasion of the mandibular condyle, making it necessary to disarticulate the condyle itself and lengthen the final bone segment while maintaining the patient-specific plate. In another case, it was necessary to readjust the reconstructive program due to interference of the skin perforator vessel with the cutting guide of the fibula. Out of the 20 patients with confirmed bone invasion, the pathological report showed free bone margins in all patients (distance range 5–10 mm); in this group, the soft tissue margins were negative (>5 mm) in 14 cases (70%), close (= 5 mm) in five cases (25%) and positive in one case (5%) (Table 2).

Post-operative concurrent chemoradiation was indicated in 15 cases (57.7%), showing single/multiple lymph nodes metastasis on the specimen with extranodal extension. Post-operative radiotherapy alone was performed in eight patients (30.8%) (seven patients staged pT4aN0; 1 patient staged pT3N0 with close soft tissue margins). Chemotherapy alone was performed in only one patient (3.8%) with mandibular metastasis from breast cancer. Two patients (7.7%) (patient no 2 and 8) did not undergo adjuvant therapy.

The follow-up period was 16–84 months. To date, 15 patients (57.7%) are alive with no evidence of disease; 8 (30.8%) patients died of disease; 3 (11.5%) patients died from other causes.

All patients underwent the same clinical and radiological follow-up: clinical visit every 3–4 months including clinical examination and videolaryngoscopy, maxillofacial and neck MRI/CT scan every 6 months, total body PET scan once a year.

#### (Group B)

In all 21 patients, the mandibular reconstruction was carried out using a osteo-fascio-cutaneous FFF and a reconstructive plate, bended manually with the aid of stereolithographic model. The average time elapsed between the CT scan and the delivery of the stereolithographic model was 8–10 days. The average time from CT scan to surgery was 25.4 days.

OCSCC was confirmed in all patients. On soft tissue negative margins (> 5 mm) were obtained in 15 cases (71.4%), close (= or <5 mm) in 6 cases (28.6%). Periosteal and bone invasion were histologically confirmed in all 21 patients. Fifteen patients (71.4%) showed an infiltrative pattern and 6 (28.6%) an erosive pattern at imaging (Table 4).

**Table 4:** Histological data for the Group B (21 patients).

***N***	**cTNM (TNM VIII Eds)**	**pTNM (TNM VIII Eds)**	**Virtual surgical planning technique**	**Type of reconstruction**	**Resection margins**	**Vascular embolization**	**Perineural invasion**	**Bone invasion**	**Bone margins**	**Periosteal margins**	**Soft tissue margins**	**Radiological pattern**	**Adjuvant treatments**	**F-UP (96–180 months)**
1	rT4aN3b	ypT4aN2b	Stereolitographic model	FFF + MRP	Negative	Yes	Yes	Yes	Negative	Negative	Negative	Infiltrative	None	DWD
2	cT4aN1	pT4aN0	Stereolitographic model	FFF + MRP	Negative	No	No	Yes	Negative	Negative	Negative	Infiltrative	RT	NED
3	rT4aN0	ypT4aN0	Stereolitographic model	FFF + MRP	Negative	Yes	Yes	Yes	Negative	Negative	Close (2 mm)	Infiltrative	None	DWD
4	rT4aN0	ypT4aN0	Stereolitographic model	FFF + MRP	Negative	No	No	Yes	Negative	Negative	Negative	Erosive	None	NED
5	cT4aN0	pT4aN3b	Stereolitographic model	FFF + MRP	Close	Yes	Yes	Yes	Positive	Negative	Close (2 mm)	Infiltrative	CRT	DWD
6	rT4aN0	ypT4aN1	Stereolitographic model	FFF + MRP	Negative	Yes	Yes	Yes	Negative	Negative	Negative	Infiltrative	CRT	NED
7	cT4aN2a	pT4aN3b	Stereolitographic model	FFF + MRP	Close	Yes	Yes	Yes	Negative	Negative	Negative	Infiltrative	CRT	DWD
8	cT4aN1	pT4aN3b	Stereolitographic model	FFF + MRP	Close	Yes	Yes	Yes	Negative	Negative	Negative	Infiltrative	CRT	DWD
9	cT4aN1	pT4aN3b	Stereolitographic model	FFF + MRP	Negative	Yes	Yes	Yes	Negative	Negative	Close (1 mm)	Infiltrative	CRT	DWD
10	cT4aN0	pT4aN0	Stereolitographic model	FFF + MRP	Negative	Yes	Yes	Yes	Negative	Negative	Negative	Erosive	CRT	DOD
11	cT4aN0	pT4aN0	Stereolitographic model	FFF + MRP	Negative	Yes	No	Yes	Negative	Negative	Close (1 mm)	Infiltrative	CRT	NED
12	rT4aN0	ypT4aN0	Stereolitographic model	FFF + MRP	Negative	Yes	Yes	Yes	Negative	Negative	Close (2 mm)	Erosive	RT	DOD
13	cT4aN1	pT4aN3b	Stereolitographic model	FFF + MRP	Negative	Yes	Yes	Yes	Positive	Negative	Close (1 mm)	Infiltrative	CRT	DWD
14	cT4aN3b	pT4aNeb	Stereolitographic model	FFF + MRP	Negative	No	No	Yes	Negative	Negative	Negative	Infiltrative	CRT	NED
15	cT4aN0	pT4aN0	Stereolitographic model	FFF + MRP	Close	Yes	Yes	Yes	Negative	Negative	Close (1 mm)	Erosive	RT	DOD
16	rT4aN0	ypT4aN0	Stereolitographic model	FFF + MRP	Close	Yes	Yes	Yes	Negative	Negative	Close (1 mm)	Infiltrative	CRT	DWD
17	cT4aN2a	pT4aN3b	Stereolitographic model	FFF + MRP	Close	Yes	Yes	Yes	Negative	Negative	Close (1 mm)	Erosive	CRT	DWD
18	cT4aN3b	pT4aN3b	Stereolitographic model	FFF + MRP	Negative	Yes	Yes	Yes	Negative	Negative	Close (2 mm)	Infiltrative	CRT	DWD
19	cT4aN0	pT4aN0	Stereolitographic model	FFF + MRP	Negative	Yes	No	Yes	Negative	Negative	Negative	Infiltrative	CRT	NED
20	rT4aN3b	ypT4aN3b	Stereolitographic model	FFF + MRP	Negative	Yes	Yes	Yes	Negative	Negative	Close (2 mm)	Erosive	CRT	DWD
21	rT4aN0	ypT4aN0	Stereolitographic model	FFF + MRP	Negative	Yes	Yes	Yes	Negative	Negative	Close (1 mm)	Infiltrative	None	DOD

*FFF, free fibula composite flap (bone + soft tissue); MRP, manually pre-shaped reconstructive plate on stereolithographic models; CRT, concurrent chemoradiation; RT, radiotherapy; NED, non evidence disease; DWD, dead for disease; DOD, dead for other disease*.

Also in the Group B, to prevent positive bone margins, the mean distance from radiologically visible lesion was 1.3 cm for infiltrative type (range from 1 to 1.5 cm) and 1 cm for erosive (range from 1 to 1.3 cm.). The pathological report showed free bone margins in 19 (90.5%) patients (distance range 5–10 mm) and positive ones in 2 cases (9.5%). The soft tissue margins were negative (>5 mm) in 10 cases (47.6%), close (< o = 5 mm) in 11 cases (52.4%) (Table 4).

Post-operative concurrent chemoradiation was indicated in 14 cases (66.7%), showing single/multiple lymph nodes metastasis on the specimen with extranodal extension or positive bone margins. Post-operative radiotherapy alone was performed in three patients (9.5%). Four patients (19%) (patient no 1, 3, 4, and 21) did not undergo adjuvant therapy.

The follow-up period was 96–180 months. To date, six patients (28.6%) are alive with no evidence of disease; 11 (52.4%) patients died of disease; 4 (19%) patients died from other causes.

All patients underwent the same clinical and radiological follow-up, similar to that performed by the patients of group A.

## Discussion

Mandibular invasion from OCSCC is one of the criteria identifying the most advanced T stage according to the American Joint Committee on cancer classification. The key elements leading the virtual programming phase are the clinical and radiological aspects of the lesion. The radiological erosive or infiltrative aspect [14] of the tumor, the extent of medullary invasion and involvement of the inferior alveolar nerve are all aspects to be considered, influencing the planning criteria for the mandibulectomy and its extent. The infiltrative type of bone involvement is characterized by significantly higher rates of positive bone margins and local recurrence (DFS 3 years = 30%) in comparison to the erosive type (DFS 3 years = 70%) [14]. Medullary invasion could contribute significantly more to poor outcome than cortical invasion, representing an independent prognostic factor in OCSCC patients [15]. When a segmental mandibulectomy is required, a precise and functional reconstruction of the mandible can be achieved most often with a FFF [9, 16–20]. The benefits are well-known and related to function/contour restoration and, when possible, to dental rehabilitation [20–24], to improve the patients' residual quality of life [8, 21].

Mandibular reconstruction with free flaps and customized plates was certainly considered to be an important step forward in recent years, especially after the advent of CAD/CAM, stereolithographic models, and virtual surgical planning. The positive impact of these technologies is certainly characterized by greater intraoperative precision and a reduction in surgical time [9, 10, 22, 23], but also by the improved functional rehabilitation of the treated patients [8]. Thanks to virtual surgical planning, all of the phases of fibula modeling and insetting of the reconstructive plate can be carried out before detaching the vascular pedicle and this represents a further advantage, especially in composite flap harvesting [10]. What is currently questionable is the accuracy of definition of the resection margins in bone and perimandibular soft tissue resection, as well as the need to radically change strategy when the planned resection proves insufficient. The accurate radiological study of bone invasion and clinical/radiological assessment of periosteal invasion are the main aspects of both locoregional staging and surgical virtual planning. If there is an unexpected progression of the tumor or a limited planned resection, the surgeon may have to modify the extent of the resection intraoperatively, nullifying the virtual planning on which the reconstruction was based. This last aspect can also be influenced by the delay required to virtually plan the surgery, although different opinions can be found in the literature [22, 23]. Virtual planning, regardless of the technique, requires a thin-layer CT scan of the head and neck and of the lower limbs. Optimization of the radiological diagnostic path is therefore of paramount importance to reduce the time to surgery.

This retrospective case series with virtually planned mandibular resection/reconstruction shows that a radical resection on soft tissue was achieved in 25 of 26 cases and in all 26 cases on bone. Intraoperative enlargement of bone resection was carried out in only one case due to unexpected periosteal involvement. Therefore, thanks to proper planning, the surgical objective was achieved in most cases; however, the main aspects making it possible were different.

Comparison between the two groups (A and B) shows similar clinical outcomes in terms of resection margins, tumor recurrence and survival. There was no evidence suggesting that predetermined surgical margins compromise oncologic safety in computer-assisted head and neck reconstruction.

The virtually planned mandibular resection/reconstruction can represent a valid tool in reducing surgery time and the burden for intra-operative decision, enhancing the accuracy of reconstruction, and increasing predictability and repeatability of surgery.

The first aspect is optimization of the radiological path. When a primary reconstruction is considered, an immediate and simultaneous request for head and neck and lower limb CT scans (both thin slice) is mandatory. If this is achieved, the time between the start of programming and surgery is acceptable.

Second, the virtual planning always takes place after completing the clinical diagnostic work-up and with the possibility to re-evaluate the patient immediately before or during the web meeting so as to implement the most precise planning without excessive bone resection. In addition, virtual planning should include a portion of residual mandibular bone >3 cm from the osteotomy to fix the plate and should also define the type of reconstruction in terms of the number of bone segments (segment length not <2.0 cm). Implant virtual planning during CAMR must also be considered an integral part of the reconstruction [24–27] so that fibular segments are placed in the best position [8].

**Figure 3:**
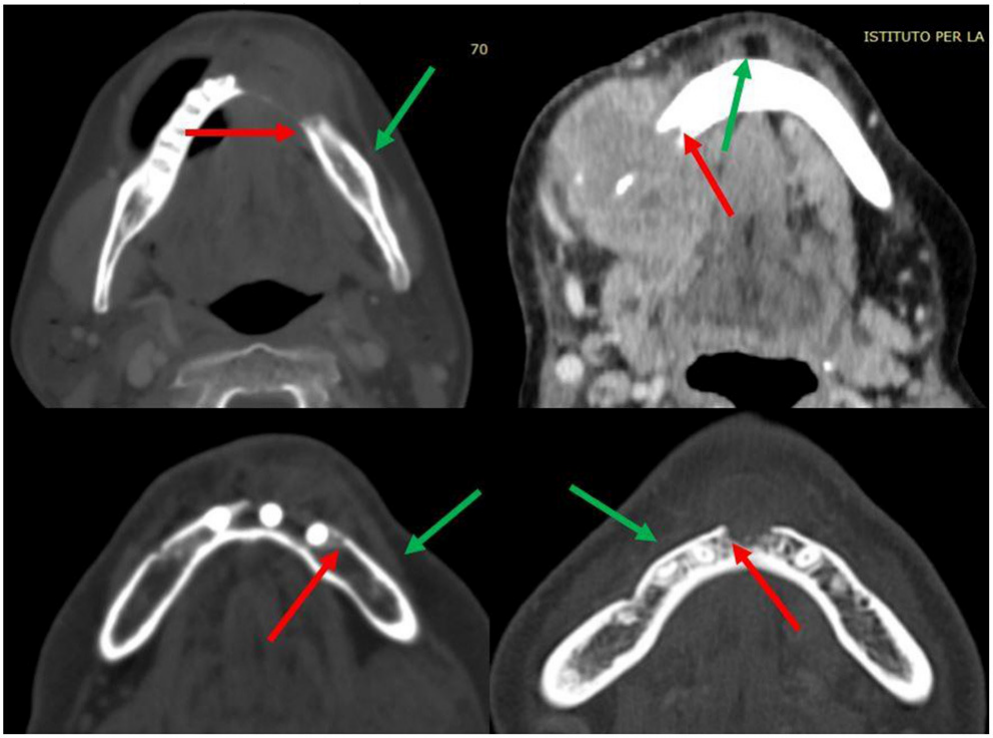
Details of some periosteal invasion and reactivity (green arrows); limit of radiologically visible bone invasion (red arrows).

Moreover, mandibular osteotomies are always planned at least 1 cm distant from the radiologically visible bone lesion when the clinical/radiological evaluation of the periosteum does not give different indications. The osteotomy is planned by adding a further 1 cm to the distance from any suspected periosteal invasion. Considering that periosteal involvement also affects the position of the mandibular cutting guides, a careful clinical-radiological evaluation is necessary (Figure 3).

**Figure 4:**
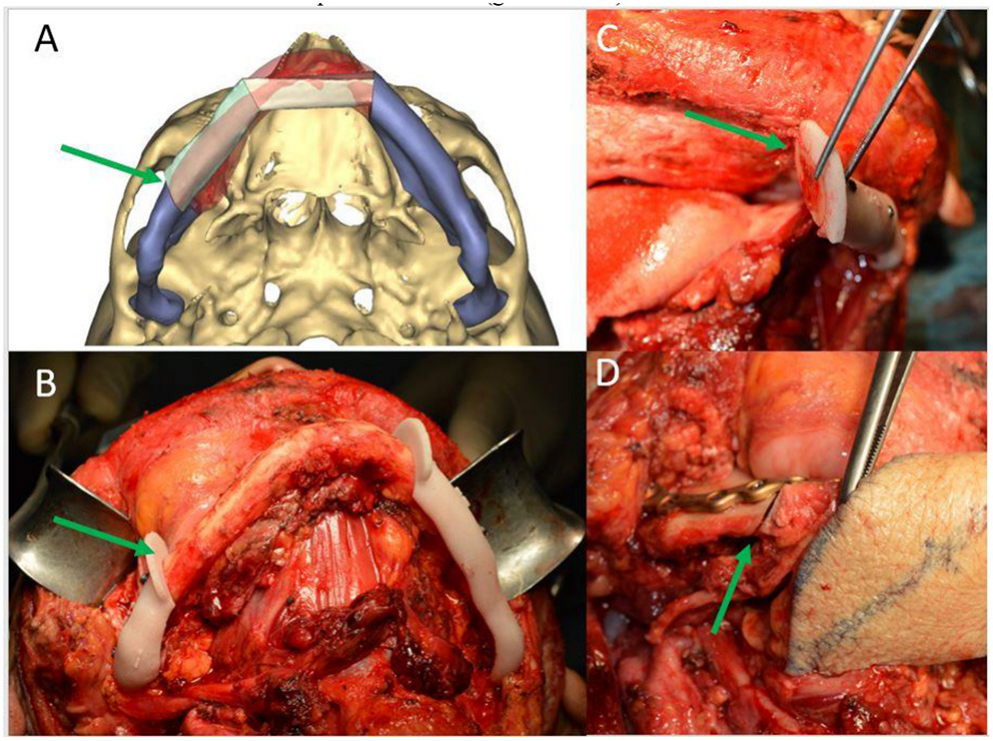
**(A)** CAMR virtual resection and reconstruction program with oblique osteotomies (green arrow); **(B)** mandibular cutting guides fixed on the mandible for oblique osteotomy (green arrow); **(C)** detail of the precision of oblique osteotomies (green arrow); **(D)** detail of the optimal adaptation of the bone surfaces after oblique osteotomies (green arrow).

According to the tumor localization (lingual or buccal), the osteotomy lines have always been planned obliquely; this strategy allows the radicality to be increased, even more than the chosen osteotomy line (0.5–1 cm) at the level of the side closest to the tumor, and to improve the contact surface between the native mandible and the reconstructed bone segment [28] (Figure 4).

Finally, synchronization of surgical times is fundamental. This last aspect has a positive effect on speed of the operation. In this series, flap harvesting was performed at the same time as the resection, to optimize the operating times. The fibula cutting guide is normally fixed and often adjusted to the position of the perforating vessels for the skin; before proceeding to the osteotomies, it was always considered that the oral mandibular resection was complete and clinically safe.

The potential discrepancy between actual tumor involvement and the virtually planned resection invokes the need to readjust the components of the virtual planning or alternatively to proceed to prepare new manually shaped plates before extending the bone resection. To anticipate an eventual “plan B,” a series of measures are necessary involving all phases of planning:

1. In the case of extensive lesions at the bone level, extend the virtual plan of the patient-specific plate (length and number of holes); if intraoperatively the lesion is considerably more extensive it becomes necessary to manually bend a new mandibular reconstruction plate, allowing the customized plate to be used as a pre-plating plate for the repositioning of the mandibular segments [27].
2. Always having a sterile 3D stereolithographic model remains a safety requirement regardless of the surgical planning performed;
3. Perform the detachment of the fibular flap only when the mandibular resection has been completed, and the bone margins are clinically safe;
4. If the extent of the resection is < 0.5 cm from the previously planned mandibular osteotomy, the customized plate (previously planned with greater extension) can allow the adaptation of a longer segment of fibula thanks to locking screws. This adaptation slightly affects the reconstructive precision by creating a gap between the plate and the bone segment itself. In this case, the cutting guide for the fibula is made to slide by 0.5 cm to perform an osteotomy compatible with the extent of the mandibular osteotomy.

To date few authors have taken into consideration the analysis and study of resection margins in the field of virtually planned surgery in head and neck oncology. Pu et al. [29], first, have compared the resection margin, recurrence pattern and survival outcomes with or without predetermined surgical margins in head and neck reconstruction. The authors concluded that predetermined surgical margins do not compromise oncological safety in terms of resection margin, disease recurrence and patient survival.

Techniques described in this article may be considered a valuable aid in the planning of composite flaps using virtual surgery, nevertheless the use of this technology requires a learning curve [30] in order to make the most of the potential and benefits of the method and at the same time to obtain free bone margins.

However, we agree with Deek and Wei [31] that computer-aided surgery is an important but limited tool, because of the many variables inherent in complex reconstruction that are not yet planned into the computer algorithm. Osteo-septo-cutaneous fibular flap components, including the septo-cutaneous vessels and the intercomponent relationship, bone cross-section and nutrition, pedicle length, and surgical plan flexibility are all important aspects to be considered during virtual planning of surgery.

## Conclusion

Virtual planning is certainly a sensitive procedure prior to mandibular reconstruction that must be approached methodically, optimally in the presence of the patient. Despite some aspects discussed here which are useful for minimizing the risks, it is always essential to be organized and able to switch quickly to an alternative intraoperative procedure. The functional benefits, the reduction in operating time, and the precision obtained in reconstruction (as demonstrated by the same authors in previous articles) seem to justify the risks to find eventually bone positive margins. During virtual planning, surgeons must bear in mind that an unexpected progression of the tumor or a limited planned resection will entail modifying the extent of the resection intraoperatively, nullifying the virtual programming on which the reconstruction was based.

## Data Availability Statement

The raw data supporting the conclusions of this article will be made available by the authors, without undue reservation.

## Ethics Statement

Ethical review and approval was not required for the study on human participants in accordance with the local legislation and institutional requirements. The patients/participants provided their written informed consent to participate in this study. Written informed consent was obtained from the individual(s) for the publication of any potentially identifiable images or data included in this article.

## Author Contributions

EC, GS, MB, EM, and IB: conception and design of the study. EC, GS, and BB: surgeons. FD'A, MT, and MB: data collection. EC and MB: paper draft. GS and MB: surgical virtual planning. GS: supervision. All authors contributed to the article and approved the submitted version.

## Funding

This research was funded by Regione Piemonte AD FUNCTIONEM (2019–2021), FPRC 5x1000 2016 Ministero della Salute Progetto ARDITE, Fondi Ricerca Corrente 2021, and Ministero della Salute.

## Conflict of Interest

The authors declare that the research was conducted in the absence of any commercial or financial relationships that could be construed as a potential conflict of interest.

## Publisher's Note

All claims expressed in this article are solely those of the authors and do not necessarily represent those of their affiliated organizations, or those of the publisher, the editors and the reviewers. Any product that may be evaluated in this article, or claim that may be made by its manufacturer, is not guaranteed or endorsed by the publisher.
